# Identification of the Mitochondrial Heme Metabolism Complex

**DOI:** 10.1371/journal.pone.0135896

**Published:** 2015-08-19

**Authors:** Amy E. Medlock, Mesafint T. Shiferaw, Jason R. Marcero, Ajay A. Vashisht, James A. Wohlschlegel, John D. Phillips, Harry A. Dailey

**Affiliations:** 1 Biomedical and Health Sciences Institute, University of Georgia, Athens, Georgia, United States of America; 2 Department of Biochemistry and Molecular Biology, University of Georgia, Athens, Georgia, United States of America; 3 Department of Microbiology, University of Georgia, Athens, Georgia, United States of America; 4 GRU-UGA Medical Partnership, University of Georgia, Athens, Georgia, United States of America; 5 Division of Hematology, Department of Medicine, University of Utah School of Medicine, Salt Lake City, Utah, United States of America; 6 Department of Biological Chemistry and the Institute of Genomics and Proteomics, University of California Los Angeles, Los Angeles, California, United States of America; Boston University School of Medicine, UNITED STATES

## Abstract

Heme is an essential cofactor for most organisms and all metazoans. While the individual enzymes involved in synthesis and utilization of heme are fairly well known, less is known about the intracellular trafficking of porphyrins and heme, or regulation of heme biosynthesis via protein complexes. To better understand this process we have undertaken a study of macromolecular assemblies associated with heme synthesis. Herein we have utilized mass spectrometry with coimmunoprecipitation of tagged enzymes of the heme biosynthetic pathway in a developing erythroid cell culture model to identify putative protein partners. The validity of these data obtained in the tagged protein system is confirmed by normal porphyrin/heme production by the engineered cells. Data obtained are consistent with the presence of a mitochondrial heme metabolism complex which minimally consists of ferrochelatase, protoporphyrinogen oxidase and aminolevulinic acid synthase-2. Additional proteins involved in iron and intermediary metabolism as well as mitochondrial transporters were identified as potential partners in this complex. The data are consistent with the known location of protein components and support a model of transient protein-protein interactions within a dynamic protein complex.

## Introduction

Metabolons are intracellular complexes of enzymes that facilitate channeling of substrates within a biochemical pathway[1]. These protein complexes serve multiple roles including the reduction or prevention of competition for metabolites by intersecting pathways (metabolic interference), increasing flux while keeping the overall concentration of pathway reactants low, and protecting cells from damage attributable to reactive metabolic intermediates[2]. Complexes of enzymes in the TCA cycle were first identified as forming a metabolon[3], and since complexes of enzymes in other pathways of intermediary metabolism including glycolysis[4], the urea cycle[5], corrinoid synthesis[6], and fatty acid metabolism[7] have been documented. Previously data have been presented in support of at least some substrate channeling among the terminal enzymes of heme synthesis[8, 9] although limited data currently exist that identify protein participants and the nature of their interactions.

In mammals, the greatest demand for heme synthesis is during erythropoiesis, with the second highest demand in the liver for xenobiotic detoxification, so most studies have been done with either hepatocytes or differentiating erythroid cells. The eight enzymes of the heme biosynthetic pathway in metazoans are distributed between the cytosol and mitochondria[10]. Along with the first enzyme, 5-aminolevulinate synthase (Alas), the terminal three enzymes of the heme biosynthetic pathway are localized within the mitochondria, while the enzymes which catalyze the central portion of the pathway are cytosolic. The product of the first reaction of the pathway, aminolevulinic acid, is exported from the mitochondria into the cytosol where it is converted to coproporphyrinogen III by the four enzymes porphobilinogen synthase (Pbgs), hydroxymethylbilane synthase (Hmbs), uroporphyrinogen III synthase (Uros), and uroporphyrinogen III decarboxylase (Urod). Coproporphyrinogen III is transported back into the mitochondria where the terminal three enzymes coproporphyrinogen oxidase (Cpox), protoporphyrinogen oxidase (Ppox) and ferrochelatase (Fech) convert coproporphyrinogen III into protoporphyrin IX and insert iron to make heme. With the exception of the first step in the pathway, all steps are catalyzed by homologous enzymes in all cells with the known regulation of the pathway occurring via transcription, splicing, translation and protein translocation[11, 12]. The only enzyme in the pathway that is encoded by two different genes is the first and rate-limiting step catalyzed by Alas. Two isoforms of this enzyme exist with one, Alas1, expressed in all cells, and a second, Alas2, expressed only in developing erythroid cells[13].

The first proposal that enzymes of heme synthesis may exist as a complex was made by Grandchamp et al. in 1978[14]. They suggested that the terminal three mitochondrially-located enzymes, Cpox, Ppox and Fech, may exist in a membrane-associated complex. Data in support of this was not forthcoming until over a decade later when several lines of evidence were garnered to support this hypothesis[8, 9, 15]. More recently it has also been demonstrated that the terminal enzyme of heme synthesis, Fech, interacts with proteins involved in mitochondrial iron transport[16]. In addition to the terminal enzymes, data supporting a complex of Alas2 and a beta subunit of succinyl-CoA synthetase (Sucla2) in differentiating erythroid cells have been provided by Furuyama et al.[17] with additional characterization by Bishop et al.[18].

For heme biosynthesis enzymes, two explanations can be offered to describe the in vivo value of being part of a complex. First, most intermediate substrates of the pathway are reduced porphyrinogens, which are highly reactive and readily autoxidize to porphyrins. These oxidized porphyrin products do not serve as intermediates in the synthesis of heme. Second, the pathway intermediates, including ferrous iron, can be cytotoxic if allowed to diffuse freely about the cell. This potential for cellular toxicity is realized in porphyrias, the pathological conditions resulting from abnormalities in the heme biosynthetic enzymes that lead to accumulation of metabolite intermediates. Depending upon the enzyme defect, these diseases may be acute in nature resulting in neurological disorders that can be life threatening (hepatic porphyrias), or non-acute resulting in photosensitivity from the elevation of porphyrin precursors in the circulatory system (erythropoietic porphyrias)[19].

In the current study, we have employed a variety of techniques to examine the interactions and distribution of proteins involved in heme synthesis in mammalian cells. We report that the mitochondrially-located heme synthesis enzymes Alas2, Ppox and Fech interact with each other as well as with additional protein partners, and appear to exist in a complex in the mitochondrion. These findings extend the originally proposed model for heme biosynthesis protein-protein interactions and identify new protein partners and roles for Fech in heme synthesis.

## Materials and Methods

### Vectors and cell lines

The human forms of seven of the eight heme biosynthetic enzymes were cloned into pEF1alpha FLAG biotag[20] vector (gift of Alan Cantor). To produce amino terminal FLAG tagged proteins, the human cDNAs encoding *PBGS*, *HMBS*, *UROS* and *UROD* were amplified from a human liver cDNA library (generated in house by H.A.D. at U.G.A.) and cloned using the XmaI-BamHI, BamHI-XbaI, BamHI-XbaI and AscI-XbaI sites, respectively. For expression of tagged CPOX and PPOX, the pEF1alpha vector was modified by QuikChange site directed mutagenesis (Agilent Technologies, Santa Clara, CA) to create a stop codon which abolished the XmaI site. The human cDNA for *CPOX* and *PPOX* were amplified from a human liver cDNA library (generated in house by H.A.D. at U.G.A.) and cloned into the modified vector pEF1alpha C FLAG using BsaBI and BstBI to produce a carboxy terminal FLAG tag. Since FECH possesses an amino terminal targeting sequence, the cDNA for the leader sequence (residues 1 to 61) was amplified from a human liver cDNA library (generated in house by H.A.D. at U.G.A.) and cloned upstream of the FLAG tag in the BsaBI and BstBI sites. The cDNA for the mature FECH protein (residues 62 to 423) was amplified from FEECH expression plasmid[21] and cloned downstream of the FLAG tag into the XmaI and XbaI sites to produce FECH pEF1alpha N FLAG. Variants of human FECH including M76H and E343K were constructed using the FECH pEF1alpha N FLAG vector and QuikChange site directed mutagenesis (Agilent Technologies).

DS19 Murine erythroleukemia (MEL) [22, 23] cells were used for all tissue culture experiments. MEL cells were transfected with expression vectors by electroporation and stably expressing cell lines were selected for puromycin resistance. Expression was confirmed by immunoblot analysis using anti-FLAG antibody (Sigma, St. Louis, MO). DS19 MEL cells were cultured in DMEM with 25 mM glucose, 1 mM sodium pyruvate and 4 mM glutamine (Cellgro, Manassas, VA) plus 10% FBS (Atlanta Biologicals, Flowery Branch, GA) and 1% penicillin/streptomycin (Cellgro). For MEL cell lines with stably expressing expression constructs, 5 μg/mL of puromycin (Cellgro) was included in media. For induction, 1.5% DMSO (Sigma) was included in growth media and cells were grown for 72 hours for maximal induction of heme biosynthetic enzymes[24].

### Affinity Purification and Mass Spectrometry

For affinity purification of CPOX, PPOX and FECH, stably expressing cell lines were grown to approximately 5–9 x 10^6^ cells/mL. Cells were centrifuged and resuspended in fresh media with 1.5% DMSO. At 72 hours, cells were harvested and mitochondria were isolated[25]. Isolated mitochondria were lysed in 1 mL of extraction buffer (50 mM Tris-HCl, pH 7.5, 1 mM EDTA, 150 mM NaCl and 1% Nonidet P-40) and centrifuged at 13,000 xg for 15 minutes at 4°C. The mitochondrial lysate was pre-cleared with Sepharose CL-4B (Sigma) for 1 hour at 4°C. Pre-cleared mitochondrial lysate was incubated overnight at 4°C with anti-FLAG agarose (Sigma). A protease inhibitor cocktail for mammalian cell extracts (Sigma) was added at 10 μl/mL of cell lysate in the overnight incubation. Anti-FLAG agarose was washed twice with wash buffer (50 mM Tris-HCl, pH 7.5, 1 mM EDTA, 150 mM NaCl and 0.1% Nonidet P-40) for 20 minutes at 4°C and eluted with 37.5 μg of FLAG peptide (Sigma) in extraction buffer for 30 minutes at 4°C. Eluted samples were analyzed by MudPIT mass spectrometry (MS) analysis[26] and immunoblots.

Affinity purification and analysis for the cytosolic enzymes PBGS, HMBS, UROS and UROD was similar to that of the mitochondrial enzymes, except that the cytosolic fraction was pre-cleared and used for affinity purification. The buffers used were the same as those described herein for the mitochondrial proteins except that detergent was excluded.

Assessment of expression of the tagged exogenous protein relative to endogenous levels was determined by comparing spectral counts of tagged exogenous protein and the endogenous protein from the MS analysis of the affinity purification samples. For proteins that form homodimers or homomultimers, we found equivalent amount of tagged exogenous and endogenous protein with the average ratio being 1.4±0.4. This suggests that in the differentiated state there were comparable amounts of the tagged exogenous and the endogenous protein orthologues. From all experiments an average of ~300 proteins were observed with normalized spectral abundance factor values over that of the control experiments. Pull downs using the mitochondrial heme biosynthesis enzymes resulted in a large number of identified mitochondrial proteins in the recovered pool. Criteria used to confirm interactions from the MS results was based on the number of spectral counts, the number of unique peptides recovered and the percent sequence coverage which occurred over that of the background on multiple samples and reciprocal pull downs. Initial efforts were focused on recovered proteins known to be involved in porphyrin, heme and iron metabolism. The protein interactions from this publication have been submitted to the IMEx (http://www.imexconsortium.org) consortium through IntAct[27] and assigned the identifier IM-24072.

### Immunoblots

For immunoblots, between 7.5 and 25.0 μg of total protein as assessed by BCA assays (Pierce, Rockford, IL) were loaded onto SDS-PAGE gels and transferred by Transblot semi-dry blotting (BioRad, Hercules, CA). Antibodies used included Anti-SUCLA2 (GeneTex, Irvine, CA) at a dilution of 1:2,500, Anti-ABCB7 (GeneTex) at a dilution of 1:500, Anti-ABCB10 (GeneTex) at a dilution of 1:1,000, Anti-PPOX (generated in house by H.A.D. at U.G.A.) at a dilution of 1:2,000, Anti-FECH (generated in house by H.A.D. at U.G.A.) at a dilution of 1:30,000–50,000, Anti-HSP-60 (Sigma) at a dilution 1:50,000, Anti-Cytochrome C (BD Biosciences, San Jose, CA) at a dilution of 1:5,000, VDAC-1 (Millipore, Billerica, MA) at a dilution of 1:2,000 and Anti-TIM-23 (BD Bioscience) at a dilution of 1:2,000. Secondary antibodies used were Anti-Rabbit IgG (H+L) HRP Conjugate (Promega, Madison, WI) and Anti-mouse IgG (H+L) HRP conjugate at dilutions of 1:30,000–60,000 with SuperSignal West Pico Chemiluminescent substrate (Pierce) and X-ray film or ChemiDoc imaging system (BioRad) for detection.

### Subcellular localization

Mitochondria were isolated from 72 hour induced MEL cells. Cellular fractionation was carried out using the reagent based Mitochondrial Isolation kit (Pierce). Mitochondrial samples were prepared from 4 x 10^7^ cells and pooled for subsequent steps. Sub-mitochondrial fractionation and protease protection assays were carried out as previously described[28, 29].

### Blue Native Page

Mitochondrial isolates from control induced cells were prepared as described above. Protein complexes from mitochondrial isolates were solubilized with 1% digitonin and ~25 μg of solubilized protein was loaded in 4–16% Bis-Tris gels for Novex NativePAGE Bis-Tris gel electrophoresis (Life Technologies, Carlsbad, CA). Sections of the Native PAGE gel were prepared by cutting regions of the gel relative to the protein marker. Gel sections were cut into small pieces and mixed with SDS-PAGE sample buffer. The resuspended proteins from each section were then separated by SDS-PAGE and transferred for immunoblotting.

### Porphyrin measurements

Undifferentiated wild-type and FECH overexpressing MEL cells were grown to ~ 2 x 10^9^ cells. Cells were harvested and washed in 1X phosphate buffered saline. Porphyrins and hemin levels were measured as previously described[30]. Briefly, washed cells were resuspended in water and sonicated for 12 cycles of 5-sec intervals at a 50% duty cycle using the micro tip. A 50 μL aliquot of the sonicated cells was mixed with 200 μL of extraction buffer ethyl acetate and glacial acetic acid (4:1). The phases were separated by microcentrifugation for 1 minute at maximum speed. The upper organic layer was immediately analyzed simultaneously for protoporphyrin IX, Zn-protoporphyrin IX and hemin by ultra-performance liquid chromatography (UPLC). The fluorescence detector channels were set at 404 nm excitation and 630 nm emission for the protoporphyrin IX, and 406 nm excitation and 586 nm emission for the Zn-protoporphyrin IX. Hemin exhibits no fluorescence and was quantified at its maximum absorbance at 398 nm. Hemin, Zn-protoporphyrin IX and protoporphyrin IX eluted at 2.42, 3.37 and 3.54 min, respectively, in three different yet simultaneously obtained chromatograms. The standard protoporphyrin IX fluorescence at the 630 nm emission setting was 41 times greater than at 586 nm emission, while the Zn- protoporphyrin IX fluorescence at 586 nm emission was 15 times greater than at 630 nm. For porphyrins (8–4 COOH), 80 μL of the sonicated cell homogenate was mixed with 80 μL 3 M HCl, incubated at 37°C for 1 hour and then spun in a microcentrifuge at maximum speed for 10 minutes. The supernatant was analyzed for porphyrin by UPLC.

### Statistical Analysis

Statistical analysis was carried out using an unpaired student *t*-test. A P value of 0.05 was set as the cutoff. Graphs show the mean with the error bars indicating the standard deviation.

## Results

### Cytosolic Enzymes Affinity Purification

To identify proteins that may exist as either stable or transient complexes of the cytosolic heme biosynthetic enzymes we used FLAG-tagged expression constructs of the human proteins and affinity purification followed by MS analysis. Since all constructs were expressed in mouse cells, and the sequences of human and mouse proteins are sufficiently different to identify each within a single sample, it was possible to identify homodimeric enzymes composed of one human subunit in complex with one mouse subunit. For each human form of the cytosolic enzymes, with the exception of UROS, the mouse form of the enzymes was recovered at high levels in the samples, but not in the control (Table 1). This is consistent with PBGS and UROD functioning as homomultimers or homodimers, and thus served as an internal control in all experiments. Affinity purification of FLAG-UROS does not result in the recovery of mouse uros since UROS is a monomer[31]. Interestingly, FLAG-HMBS resulted in the recovery of a significant number of peptides of the mouse form of the enzyme. While this enzyme is reported to function as monomer, the human enzyme crystallized as two monomers in the asymmetric unit with a weak dimer interface[32]. Results from our studies suggest that HMBS in differentiating erythroid cells may exist in vivo as a homodimer. The amino terminal FLAG-PBGS, -HMBS, -UROS and-UROD did not result in the recovery of any other cytosolic heme biosynthetic enzyme suggesting that if a cytosolic heme biosynthetic metabolon forms, it is not via direct interactions between enzymes, or that these interactions are weak and not stable to our isolation procedure. Although no direct interactions or common core proteins were observed it is possible that the cytosolic enzymes cluster to facilitate substrate channeling[33].

**Table 1:** Affinity Purification and MS results of Cytosolic Heme Biosynthesis Enzymes.

	Human Input				
	PBGS	HMBS	UROS	UROD	Empty vector
	(n = 2)	(n = 3)	(n = 3)	(n = 3)	(n = 3)
**Murine protein**					
**pbgs**	0–325	0	0	0	0
	0–17	0	0	0	0
	0–39%	0%	0%	0%	0%
**hmbs**	0	0–383	0	0	0
	0	0–7	0	0	0
	0%	0–11%	0%	0%	0%
**uros**	0	0	0	0	0
	0	0	0	0	0
	0%	0%	0%	0%	0%
**urod**	0	0	0	288–868	0–6
	0	0	0	10–50	0–5
	0%	0%	0%	8–65%	0–16%

Data shown from top value to bottom as: spectral counts/ unique peptides/ percent sequence coverage.

### Mitochondrial Enzymes Affinity Purification and Immunoblot

**Fig 1:**
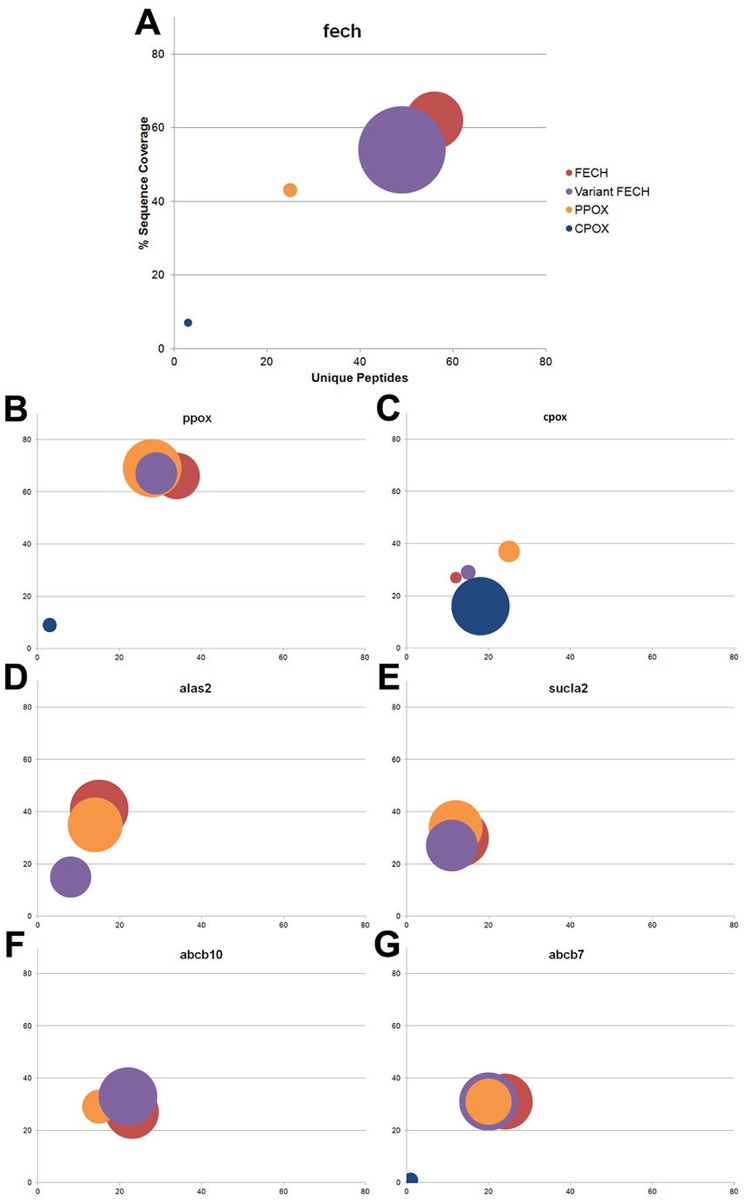
Graphical representation of affinity purification and MS analysis of FLAG-FECH (red), FLAG-FECH Variants (purple), FLAG-PPOX (orange) and FLAG-CPOX (blue). Each panel represents an identified mouse protein recovered with the bait human protein listed in the legend of panel A. Panels are as follows: (A)—fech, (B)—ppox, (C)—cpox and (D)—alas2, (E)—sucla2, (F)—abcb10 and (G)—abcb7. Number of unique peptides (x axis), % sequence coverage (y axis) and spectral counts (bubble size) for each was calculated using the maximal values obtained minus the maximal values observed in the control samples (empty vector). Size of bubbles represents the % of the total spectral counts identified for each mouse protein. The maximal spectral counts of each of the mouse proteins was fech = 971, ppox = 71, cpox = 256, alas-2 = 16, sucla2 = 16, abcb10 = 51 and abcb7 = 36. Note no mouse peptides for alas2, sucla2, or abcb10 were obtained using FLAG-CPOX as bait above that of empty vector.

**Fig 2:**
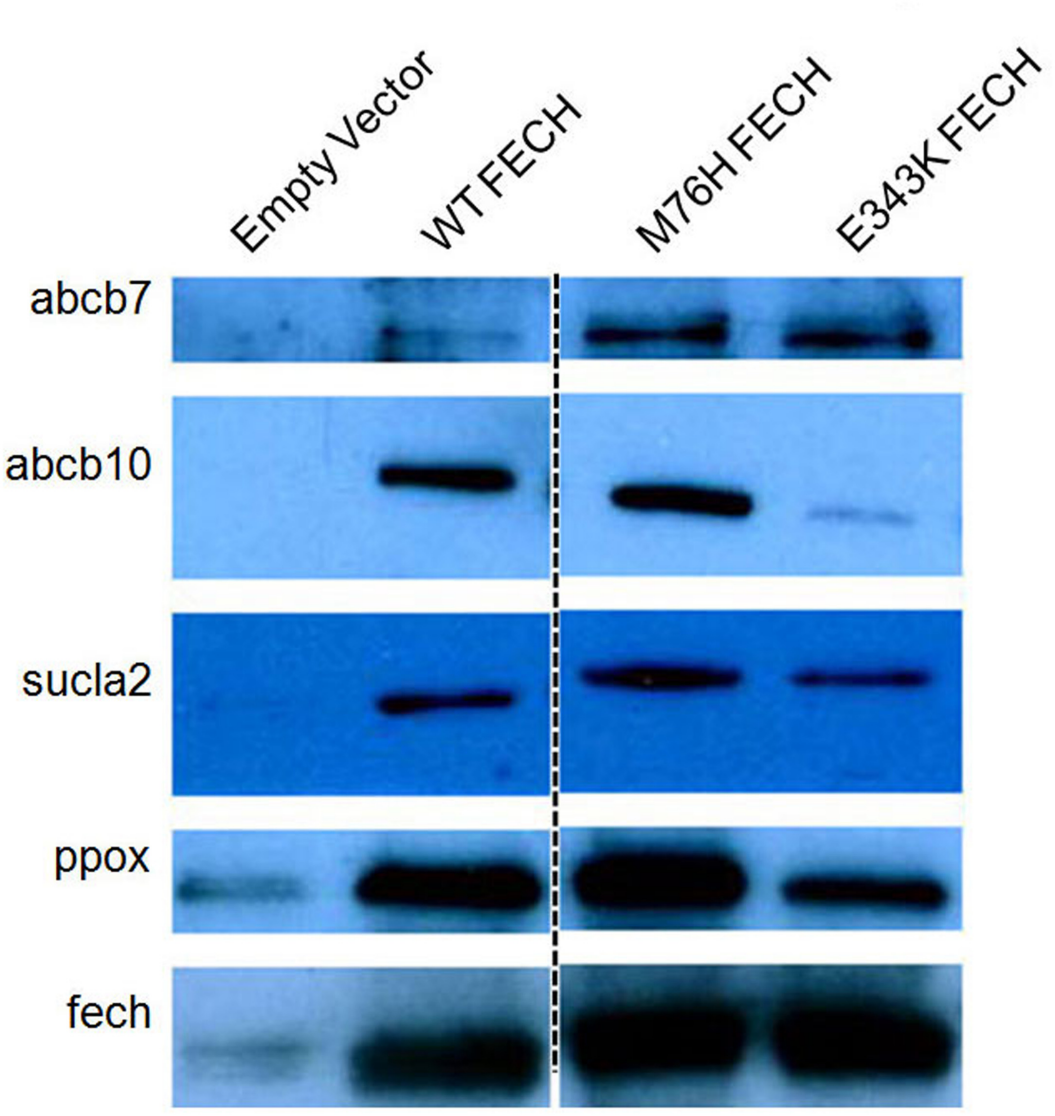
Immunoblot from Affinity Purification of FLAG elutions. Each lane represents the FLAG elutions from the affinity purification using empty vector (lane 1), WT FECH (lane 2), M76H FECH variant (lane 3) and E343K FECH variant (lane 4). Blots were probed for abcb7, abcb10, sucla2, ppox and fech. The dashed line indicates non consecutive lanes on the same gel.

Previous kinetic and isotope labeling studies[8, 9] along with in silico structural studies[15] have proposed a complex between the terminal enzymes, CPOX, PPOX and FECH to facilitate substrate channeling. For our approach to identify a mammalian mitochondrial heme biosynthesis complex, affinity purification and MS of the tagged versions of the terminal three enzymes were carried out. As observed with the cytosolic enzymes, CPOX, PPOX and FECH all recovered the mouse form of the enzymes at high levels with the tagged human bait. This is consistent with CPOX, PPOX and FECH being functional homodimers. With FLAG-FECH as bait, the data support interactions between FECH and ppox, but not between FECH and cpox (Figs 1 and 2 and Table 2). These results were validated by performing the inverse experiment, eg. FLAG versions of PPOX and CPOX were used as bait. The data from these experiments support the affinity purification data with FLAG-FECH (Fig 1 and Table 2). It is important to note that FECH, PPOX and CPOX are all membrane associated proteins and thus “sticky” in nature, so even in the negative controls these proteins were detected at low levels. While an interaction between PPOX and CPOX may still occur via a transporter or bridge protein, identification of this protein could not be confirmed from our results. Proteins previously identified as porphyrin transporters, including transmembrane protein 14c (tmem14c)[30], 2-oxoglutarate/malate carrier (ogc)[34] and adenine nucleotide translocators (ant1/2)[35] were observed (Table 2) and may function as bridging proteins and participate in protoporphyrinogen transport.

**Table 2:** Affinity Purification and MS results of Mitochondrial Heme Biosynthesis Enzymes.

	Human Input				
	FECH	FECH variants	CPOX	PPOX	Empty Vector
	(n = 2)	(n = 3)	(n = 3)	(n = 2)	(n = 2)
**Murine Protein**					
**fech**	297–443	195–997	27–35	38–52	9–26
	29–63	39–56	6–10	10–32	4–7
	46–79%	48–71%	15–24%	23–60%	13–17%
**ppox**	20–47	24–39	4–9	33–71	2–7
	11–37	16–32	3–6	9–31	2–3
	26–75%	45–76%	7–18%	22–78%	7–9%
**cpox**	20–19	10–26	89–256	3–44	5–10
	9–19	10–22	16–25	3–32	4–7
	24–50%	24–52%	31–39%	10–60%	12–23%
**alas-2**	0–16	3–8	0	0–14	0
	0–15	3–8	0	0–14	0
	0–41%	5–15%	0	0–35%	0
**sucla2**	9–16	8–13	0–5	3–14	0–2
	8–15	8–13	0–5	3–14	0–2
	20–37%	20–34%	0–17%	6–41%	0–7%
**abcb10**	30–38	24–51	3–8	3–24	3–10
	16–29	22–28	3–5	3–21	2–6
	28–45%	39–44%	6–9%	4–40%	4–11%
**abcb7**	17–33	18–36	2–6	0–24	0–4
	8–27	17–23	2–4	0–23	0–3
	12–37%	21–37%	4–7%	0–37%	0–6%
**ADP/ATP translocase 1 slc25a4 (ant1)**	21–75	23–152	9–28	24	3–13
	12–16	15–26	5–9	13–14	2–4
	28–48%	41–53%	14–27%	30–49%	6–7%
**ADP/ATP translocase 2 slc25a5 (ant2)**	52–111	41–228	14–43	28–34	9–13
	18–23	19–26	6–15	13–16	4–7
	31–65%	49–71%	19–38%	26–47%	12–13%
**voltage dependent anion selective protein channel 2 (vdac-2)**	4–11	6–14	0–6	4–12	0
	3–10	6–11	0–3	4–11	0
	11–43%	28–43%	0–16%	20–43%	0%
**2-oxoglutarate/malate carrier slc25a11 (ogc)**	6–14	7–27	0–4	12–13	0–2
	4–14	7–14	0–2	3–12	0–2
	15–43%	24–42%	0–8%	12–45%	0–8%
**transferrin receptor protein 1 (tfrc)**	12–25	23–31	0	0–22	0–2
	11–24	23–25	0	0–22	0–2
	13–37%	32–37%	0%	0–32%	0–3%
**sideroflexin-1 (sfxn1)**	0–7	3–9	0	0–11	0
	0–7	3–9	0	0–11	0
	0–26%	12–44%	0%	0–45%	0%
**metalloreductase steap3 (steap3)**	0–3	2–6	0	0–3	0
	0–3	2–6	0	0–3	0
	0–7%	4–14%	0%	0–7%	0%
**glutaredoxin 5 (glrx5)**	4–6	2–10	0–2	0–3	0
	3–4	2–3	0–2	0–3	0
	22–47%	22%	0–22%	0–22%	0%
**transmembrane protein 14C (tmem14c)**	0	0	0	0–4	0
	0	0	0	0–4	0
	0%	0%	0%	0–50%	0%

Data shown from top value to bottom as: spectral counts/ unique peptides/ percent sequence coverage.

Interestingly, in addition to the interaction between PPOX and FECH, the mitochondrial matrix-located first enzyme in the pathway, alas2, and its previously identified[17, 18] protein partner sucla2 were both observed when either FECH or PPOX were used as bait (Fig 1 and Table 2). Interaction between SUCLA2 and ALAS2 was postulated to be part of a complex that forms to provide succinyl-CoA for aminolevulinic acid synthesis and prevent succinyl-CoA from being consumed by the TCA cycle[17], although no biochemical or kinetic data exist to support this model. The interaction between ALAS2, FECH and PPOX has not previously been proposed and thus was an unexpected finding. Interaction between FECH and sucla2 was validated via immunoblot (Fig 2), but validation of the interaction with alas2 was not possible due to low quality of available antibodies for alas2.

Several proteins proposed to be involved in mitochondrial iron metabolism, including abcb10 and abcb7, were identified in the FLAG FECH affinity purification experiments (Figs 1, 2 and Table 2). Interactions between FECH and Abcb10[16] or Abcb7[36] have been previously shown and were further validated. Immunoblot analysis of FLAG FECH confirmed these interactions. Interestingly, mitoferrin, the protein previously identified as being an erythroid-specific iron transporter for heme synthesis[37], was not identified from the FLAG FECH experiments. This may be explained by weak or transient interactions between FECH and mitoferrin or by bridging interactions between proteins such as Abcb10 or Abcb7. The affinity purification experiments of FLAG mitoferrin resulted in the isolation of only a small number of fech spectral counts and unique peptides[16], thus subtle differences in induction and/or purification may explain our result. Several other proteins involved in iron metabolism including glutaredoxin 5, sideroflexin-1, transferrin receptor-1 and Steap3 were also identified in the FECH affinity purification samples (Table 2). It is of note that the latter two proteins are not localized to mitochondria, therefore interaction with FECH may be during organelle contact for iron delivery via the kiss and run mechanism[38–40] and mediated by other identified bridging proteins[41]. The direct or indirect role of interactions between these proteins and FECH is unclear, but of note due to their known role in iron trafficking and homeostasis[42, 43].

Different FECH variants have been shown to assume independent, stabilized structural conformations each consistent with one of the three structural catalytic FECH intermediates[44]. We hypothesize that different protein-protein interactions may be stabilized during our affinity purification experiments with the FECH variants, however we did not observe significantly different data (Figs 1 and 2). The fact that the variant monomers form homodimers with the wild-type mouse form of fech, which is expressed at high levels during induction, may explain why no significant differences were noted. Human FECH variants M76H and E343K showed some differences in the spectral counts of protein partners, but these were not considered to be different from those of the wild-type enzyme partners (Table 2). It is possible that uninduced cells or knock down of the endogenous wild-type mouse Fech will be required to determine the differences in protein partners for human FECH variants.

### PPOX Localization

**Fig 3:**
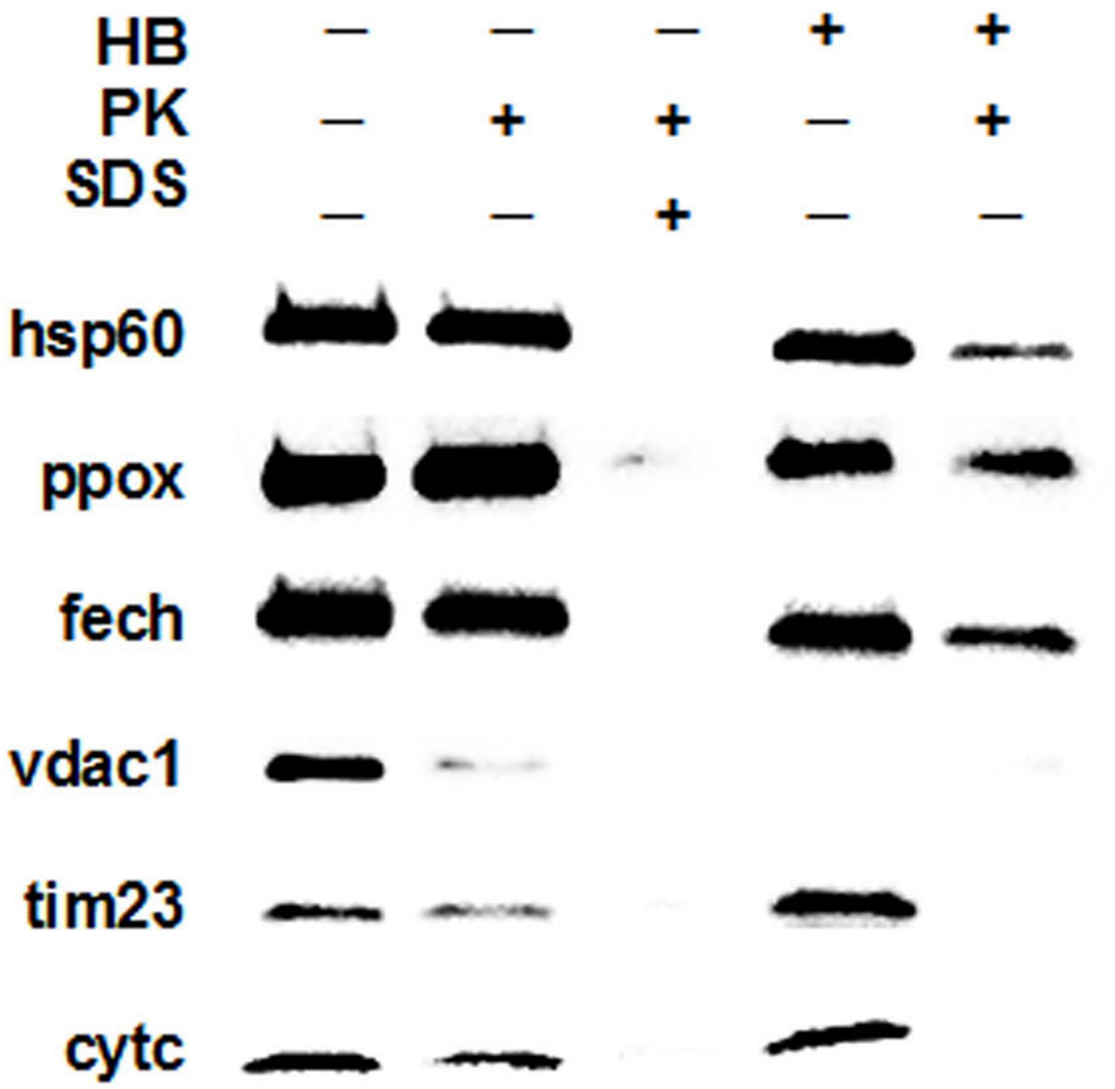
Subcellular fractionation and protease protection. 7.5 ug of mitochondrial protein was processed and loaded in each lane. HB refers to treatment with hypo-osmotic buffer (20 mM KCl) for mitoplast isolation, PK to proteinase K (1 mg/mL) treatment and SDS to sodium dodecyl sulfate (0.5% w/v) treatment for mitoplast lysis. Markers for each compartment include hsp60—matrix, vdac1—outer mitochondrial membrane, tim23—inner mitochondria membrane and cytc—intermembrane space.

In order to obtain pull-down data for putative interacting proteins, it must be clear that all participating proteins possess some physical overlap within a single cellular compartment. Previous cellular fractionation and activity data were consistent with Ppox being associated with the outer face of the inner mitochondrial membrane[8, 45] in the same space as Cpox[14, 46]. However, a recent in situ enzymatically-based chemical labeling study by Rhee et al.[47] suggested that PPOX is, in fact localized to the matrix and that CPOX, while located in the inner mitochondrial membrane space, possesses an amino terminal transmembrane segment that ends in the matrix. Because of this and our affinity purification results presented herein, we re-examined the location of ppox using classical cellular fractionation and mitoplast preparation followed by protease treatment. Results from these studies support localization of ppox to the mitochondrial matrix side of the inner membrane (Fig 3). These submitochondrial localization studies in combination with the affinity purification results suggest that a direct interaction in the mitochondrial matrix between FECH and PPOX exists and, thus, support the presence of a transporter for movement of protoporphyrinogen IX across the inner mitochondrial membrane[30, 34, 35].

### FECH-PPOX complex

**Fig 4:**
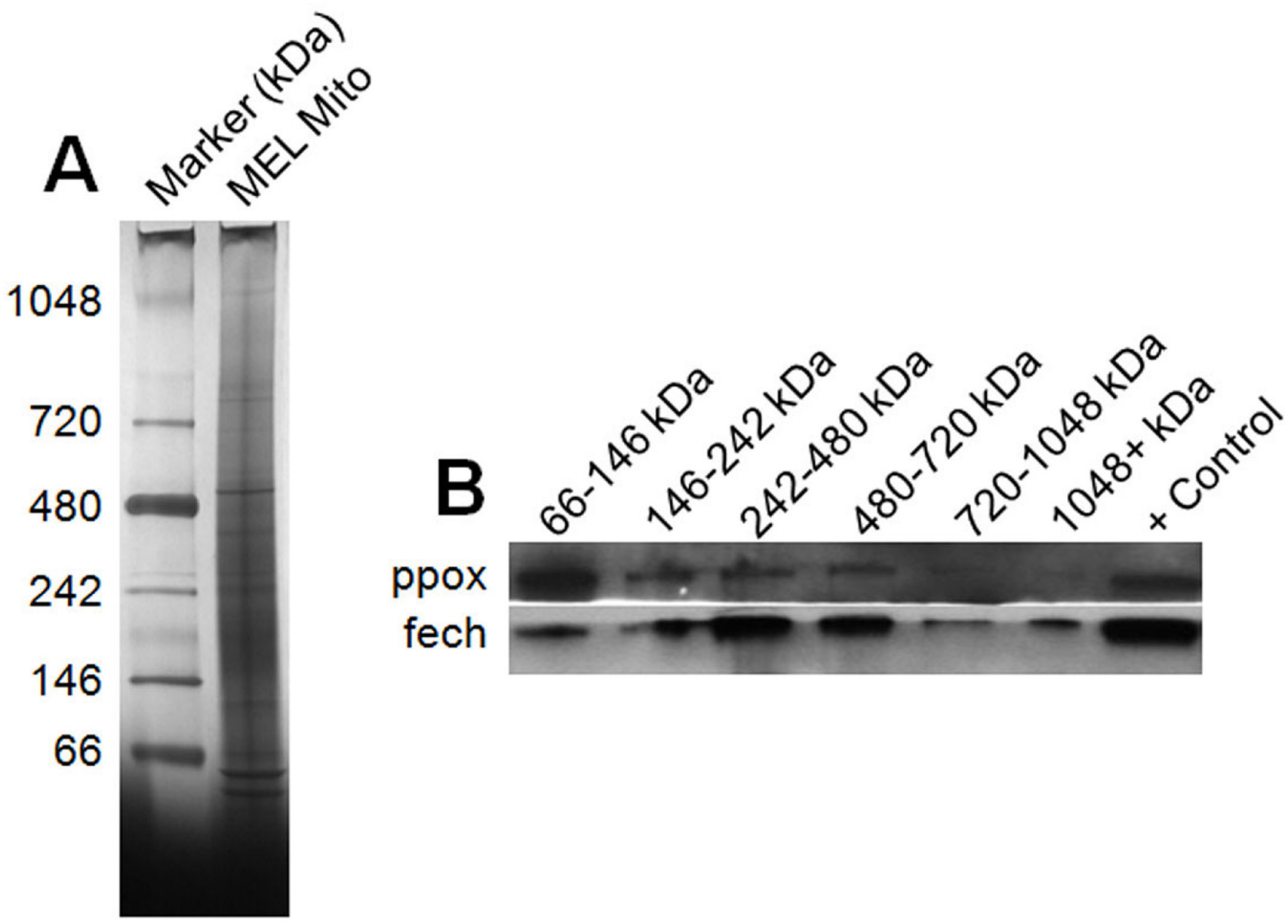
Native PAGE and Western blot analysis of mitochondrial protein complexes. (A) Solubilized proteins complexes from mitochondrial preparations of differentiated MEL cells were separated by Native PAGE. (B) Regions of the Native PAGE gel were excised and proteins further separated by SDS-PAGE. Western blot of the SDS-PAGE was carried out for ppox and fech.

To further investigate high molecular weight complexes that contain fech and ppox from mitochondria of induced MEL cells, blue native PAGE gel electrophoresis followed by SDS-PAGE and immunoblotting was carried out. Results from these experiments show some overlap between fech and ppox in complexes with estimated molecular weights ranging from 66–720 kDa based on a protein standard (Fig 4). At minimum a homodimer of ppox and a homodimer of fech would result in a complex of approximately 186 kDa. The presence of increased amounts of fech in complexes with an estimated molecular weight between 242–720 kDa supports the presence of additional proteins as observed from the affinity purification of these complexes.

### Porphyrin Levels

**Fig 5:**
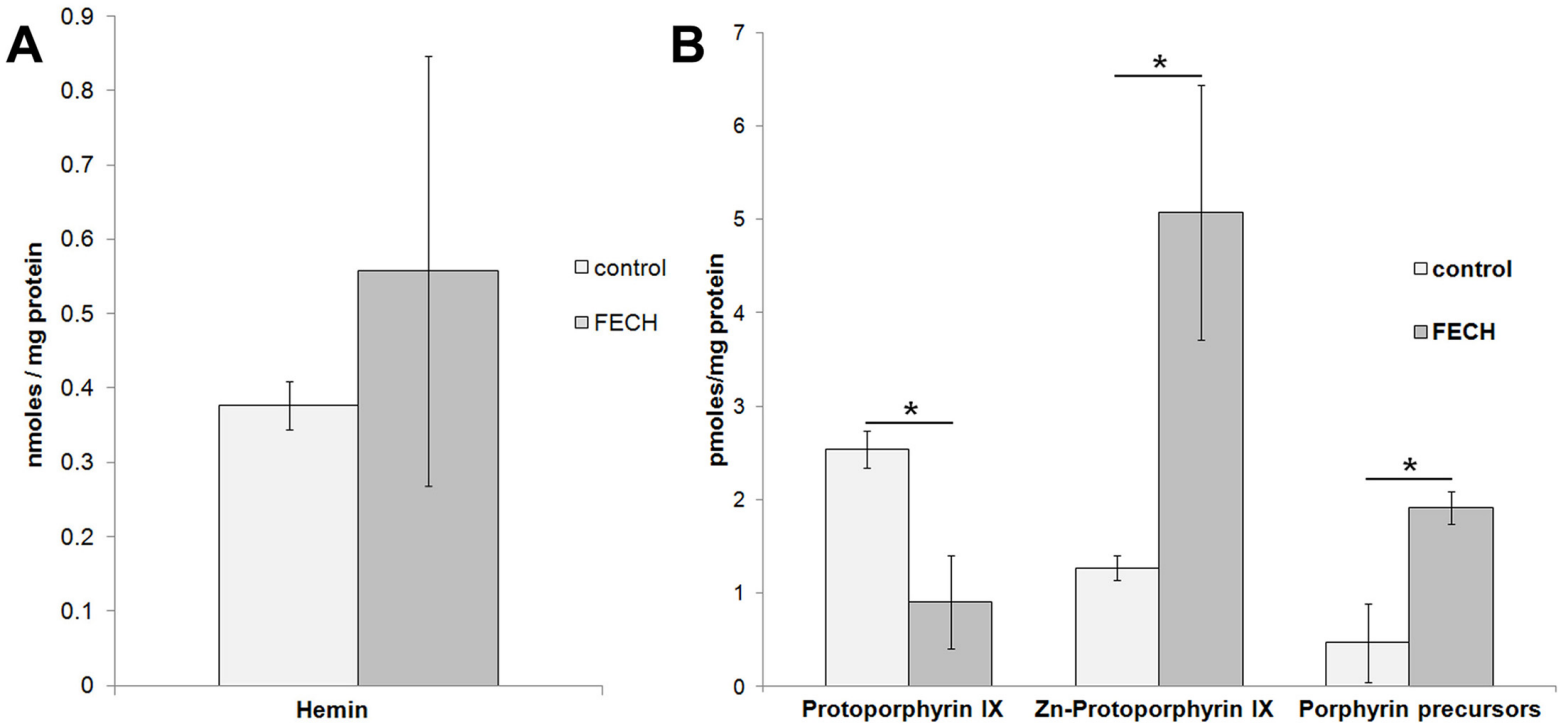
Porphyrin and metallated porphyrin levels in undifferentiated MEL cells. (A) Hemin levels from undifferentiated control (n = 3) and FECH overexpressing (n = 4) MEL cells.. (B) Levels of protoporphyrin IX, Zn-protoporphyrin IX and porphyrin precursors (8–4 COOH porphyrins) for undifferentiated control (n = 3) and FECH overexpressing (n = 4) MEL cells. * indicates a *p* value of ≤ 0.006.

The expression of exogenous tagged heme biosynthetic enzymes in our model system led us to investigate what effect altered levels of FECH may have on cellular heme production. Analysis of heme and porphyrin levels in differentiated MEL cells from both control and with exogenous FECH expression resulted in large variations in the levels of these metabolites dependent on the degree of induction of cells (data not shown). Since induction of the heme biosynthetic enzymes during MEL cell differentiation is biphasic with the terminal enzymes occurring after 48 hours[48] and therefore limiting heme synthesis[49, 50], we investigated the effect of FECH overexpression in undifferentiated cells. Undifferentiated MEL cells represent a more homogenous population of cells in which all the endogenous heme biosynthesis enzymes remain at constant levels. The intracellular levels of hemin, protoporphyrin IX, Zn-protoporphyrin IX and porphyrin precursors (8–4 COOH porphyrins) were measured in undifferentiated DS19 MEL cells and MEL cells overexpressing FLAG FECH. While the hemin levels were not different in FECH overexpressing cells (Fig 5A), the porphyrin profiles did show several differences. Specifically, the intracellular levels of porphyrin precursors and Zn-protoporphyrin IX were significantly increased, while the protoporphyrin IX levels were decreased (Fig 5B). The increase in porphyrin precursors in cells overexpressing FECH was unexpected and suggests that FECH plays a role in influencing early steps in porphyrin precursor synthesis in the mitochondria possibly via interaction with ALAS2 or by interacting with iron or heme transporters. It is plausible that interactions between FECH, PPOX, ALAS2 and SUCLA2 play a regulatory role and may explain the loss in coordination between porphyrin synthesis and iron metabolism observed in X-linked protoporphyria where mutant ALAS2 has increased activity[51].

## Discussion

The data presented herein along with previous studies[8, 9] support the existence of a mitochondrial heme metabolism complex composed of Fech, Ppox, Alas2, Abcb10, Abcb7, and Sucla2 to facilitate substrate channeling and coordinate iron and porphyrin metabolism. Proteins identified as interacting with the terminal enzyme of the heme biosynthetic pathway, Fech, include a number of proteins involved in porphyrin synthesis (Alas2 and Ppox) and iron metabolism (Abcb10 and Abcb7). These interactions stress the importance of Fech as an important regulatory point at the intersection of these two cellular pathways. It is of note that Fech was found to interact with several proteins involved in iron sulfur cluster metabolism. One such protein Abcb7 is involved in both iron and heme metabolism as mutations in Abcb7 result in sideroblastic anemia[52]. Abcb7 exports an unknown factor(s) required for cytosolic iron sulfur cluster metabolism[53]. Thus the interactions between Fech and Abcb7 could play a role in regulating the cellular distribution of iron or it may be that Abcb7 interacts with Fech as part of the assembly or stabilization of the Fech [2Fe-2S] cluster. During erythropoiesis the demand for iron is high at several levels including for the regulation of Alas-2 translation via its iron regulatory element[54, 55], thus tying the cellular distribution of iron and the production of heme together. Other benefits of such a complex for mitochondrial heme synthesis include cellular protection from reactive molecules including porphyrins, iron and heme, rapid and targeted synthesis of heme for erythropoiesis, and coordinated distribution of heme and iron. A precedent for such regulation in cofactor synthesis has been observed in vitamin B_12_ synthesis in which the pathway intermediates are unstable[6]. The formation of transient protein complexes for substrate channeling may be a common mechanism for pathways with reactive intermediates to facilitate pathway efficiency and to protect the cellular milieu from damage due to these reactive intermediates. Previous studies using coproporphyrinogen III and iron with intact mitochondria have shown that protoporphyrin intermediates do not accumulate[9] suggesting the role of the a protein complex in protection from reactive intermediates. Our data support an interaction between Ppox and Fech for the transfer of protoporphyrin. The proteins which transport protoporphyrinogen IX between the intermembrane space and the matrix, or the heme exporter, are yet to be clearly identified, but are likely inner membrane transporters, such as Tmem14c[30], Ant2[35], Ogc[34] and Abcb10[56–60]. Each of these transporters has been implicated in heme synthesis during erythroid differentiation and are suggested by our data to be components of a mitochondrial protein complex with Fech.

The large number of protein partners for Fech observed in the affinity purification and MS analysis was a surprise and highlights the complexity of the mitochondrial heme metabolism protein complex. This complexity was suggested in previous studies by Ferreira et al.[8] who showed that only in intact mitochondria could protoporphyrinogen and iron be used to produce heme independent of added of protoporphyrin at low concentrations. This was not the case in solubilized mitochondria were utilized, nor when the terminal enzyme were purified and reconstituted into phospholipid vesicles. Thus the terminal enzymes plus other proteins in intact mitochondria were necessary for heme formation. The data presented herein have identified and validated several of the players in this complex and further studies will be necessary to map the precise interactions and understand the role of each player in heme production. It is likely that only the major players in the complex have been identified and that other minor components have yet to be detected. Cross-linking may help to pull out these low abundance proteins as well as map the interactions of the major players as direct or via bridging proteins. The large number of partners identified in our study begs the question—how many protein partners can Fech have? It is unlikely that Fech interacts with multiple partners in a stable complex, but more probable the interactions between Fech and its protein partners are transient and dependent on the conformation of the enzyme. Previous structural studies have shown that Fech adopts several distinct conformations which correspond to stages of its catalytic cycle[44, 61]. These show that Fech is a dynamic enzyme that undergoes conformational changes during catalysis and that each conformation provides distinct enzyme surfaces which would stabilize interactions with partners such as Ppox for porphyrin delivery or Abcb10 and mitoferrin for iron delivery[44]. While the identification of the details of interactions of protein partners with Fech variants which exist in specific conformations was not possible with the current experimental approach, the large number of putative partners for Fech that were identified supports the proposal of multiple transient complexes.

In addition to characterization of a heme metabolism protein complex three points of interest that have significant impact were revealed in the current study. First, data were obtained that suggest the cytosolic protein Hmbs is a functional homodimer, not a monomer as presumed from in vitro studies of the purified protein[32, 62]. Second, Ppox was identified as a homodimer in situ as opposed to a monomer as suggested from in vitro studies by others[63]. Third, the location of Ppox was confirmed to be in the mitochondrial matrix[47] and not the inner membrane space. This location is critical since it supports the proposal that Tmem14c is a protoporphyrinogen, not porphyrin, transporter[30].

The matrix localization of Ppox along with the proposed orientation of Ppox and Fech[8] suggests that some mobility exists within the mitochondrial heme metabolism protein complex. With one active site per enzyme monomer by which substrate must enter and product leave, some motion must occur for entry of the porphyrinogen or porphyrin and exit of the porphyrin or heme. Clustering of enzymes which have some mobility within their environment has shown to be as efficient as direct interactions for substrate channeling[33]. One of the most well known examples in biochemistry for protein mobility and interactions is in the interaction of cytochrome c with different components of the electron transport chain. In the electron transport chain, cytochrome c acts as a mobile protein which interacts with distinct partners depending on its redox state[64]. While the mobility of proteins in the mitochondrial heme metabolism protein complex may be somewhat restricted due to the variable association of the components with the inner mitochondrial membrane, we hypothesize based on the data presented herein that there is some mobility between protein partners.

In addition to the protein partners of Fech we also investigated the effect of expression of FLAG FECH in MEL cells. Expression of exogenous FLAG FECH resulted in a functional system in undifferentiated and differentiated MEL cells which produced a comparable amount of heme relative to MEL cells with only endogenous fech. Interestingly, there were noted perturbations to porphyrin homeostasis in undifferentiated cells overexpressing FECH. This finding supports a temporal role for FECH in regulating cellular levels of porphyrins[48, 49]. The increased levels in porphyrin precursors (8–4 COOH porphyrins oxidized from porphyrinogens in cells) in undifferentiated FECH overexpressing cells suggest that FECH may stimulate early steps in precursor production or transport by serving as a nucleation site for clustering of enzymes involved within the mitochondria. The elevated level of Zn-protoporphyrin, but decreased protoporphyrin, is also an interesting and unexpected finding. The fact that heme levels do not differ suggests that iron import is limiting due to culture conditions[65, 66] or lack of machinery[37, 67] and that Zn-protoporphyrin IX may be a result of increased porphyrin synthesis without the subsequent up regulation of iron import. Thus the Zn-protoporphyrin is either formed by human FECH activity, an unknown enzyme or an enzyme independent process. Further analysis of porphyrin levels in MEL cells overexpressing non-functional variants of human FECH will provide insight into the role of Fech in porphyrin precursor synthesis and/or export and in Zn-protoporphyrin production, a compound relevant in several disease states including lead poisoning, iron deficiency anemia and X-linked protoporphyria.

**Fig 6:**
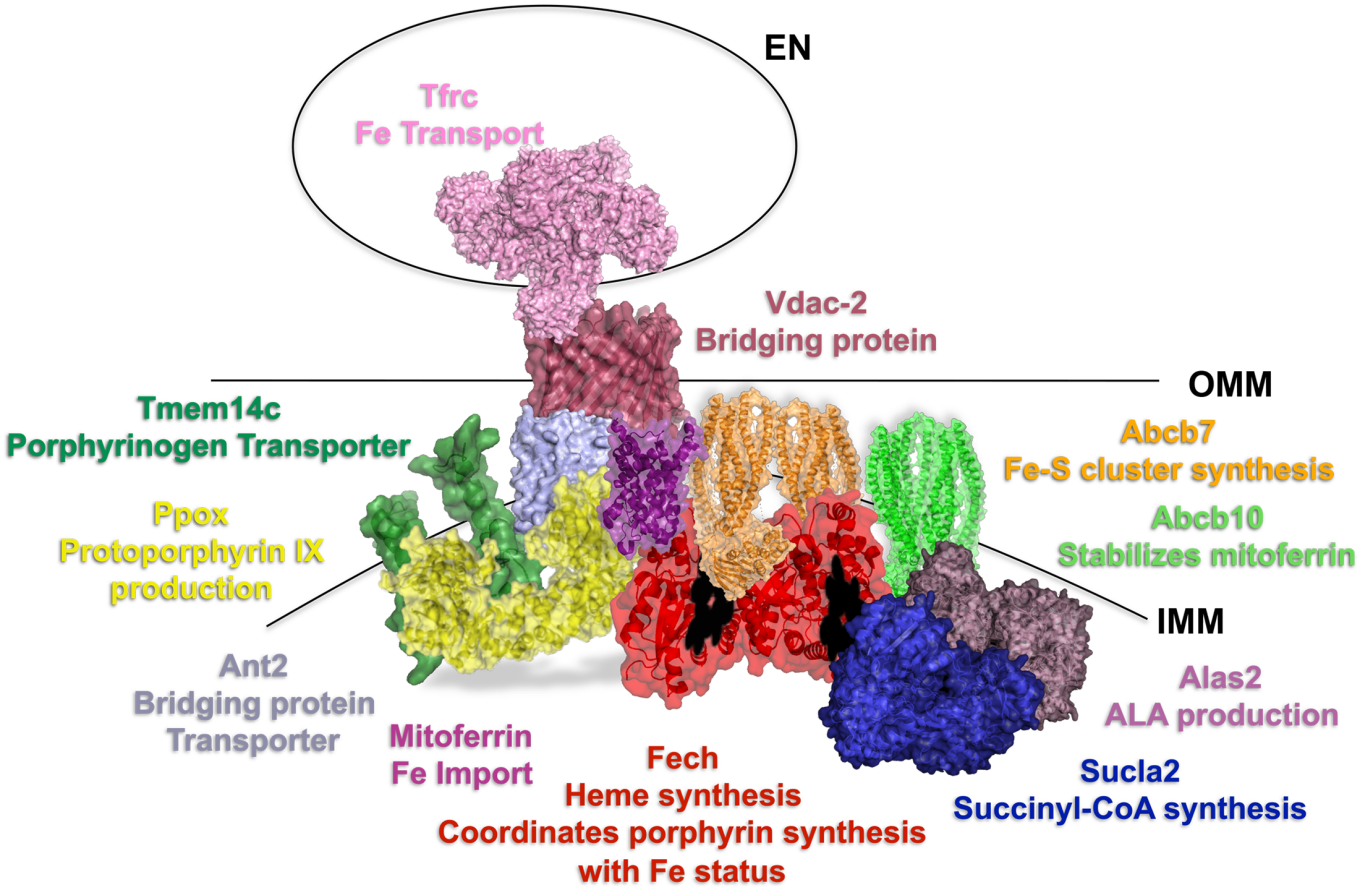
Proposed model of Mitochondrial Heme Metabolism Protein Complex. Proteins in the complex are shown in the inner mitochondrial membrane and labeled with their roles in porphyrin, iron and heme homeostasis. Additional proteins which may be involved in bridging between protein partners are not shown. IMM refers to the inner mitochondrial membrane, OMM to the outer mitochondrial membrane and EN to the endosome.

In summary, data presented herein support the interaction between several mitochondrial heme biosynthetic enzymes (Alas2 and Ppox), proteins involved in iron and iron-sulfur cluster homeostasis (Abcb10 and Abcb7), and mitochondrial transporters (Abcb10) for a mitochondrial heme metabolism protein complex (Fig 6). Further studies are necessary to define the nature of the interactions, i.e. direct or via bridging proteins, as well as the precise role of this complex in heme metabolism.
